# Mapping and Congenic Dissection of Genetic Loci Contributing to Hyperglycemia and Dyslipidemia in Mice

**DOI:** 10.1371/journal.pone.0148462

**Published:** 2016-02-09

**Authors:** Weibin Shi, Qian Wang, Wonseok Choi, Jing Li

**Affiliations:** 1 Departments of Radiology and Medical Imaging, University of Virginia, Charlottesville, Virginia, United States of America; 2 Biochemistry & Molecular Genetics, University of Virginia, Charlottesville, Virginia, United States of America; Temple University School of Medicine, UNITED STATES

## Abstract

**Background:**

Patients with dyslipidemia have an increased risk of developing type 2 diabetes, and diabetic patients often have dyslipidemia. Potential genetic connections of fasting plasma glucose with plasma lipid profile were evaluated using hyperlipidemic mice.

**Methods:**

225 male F_2_ mice were generated from BALB/cJ (BALB) and SM/J(SM) *Apoe*-deficient (*Apoe*^−/−^) mice and fed a Western diet for 5 weeks. Fasting plasma glucose and lipid levels of F_2_ mice were measured before and after 5 weeks of Western diet and quantitative trait locus (QTL) analysis was performed using data collected from these two time points. 144 SNP(single nucleotide polymorphism) markers across the entire genome were typed.

**Results:**

One major QTL (logarithm of odds ratio (LOD): 6.46) peaked at 12.7 cM on chromosome 9,*Bglu16*, and 3 suggestive QTLs on chromosomes 15, 18 and X were identified for fasting glucose, and over 10 loci identified for lipid traits. *Bglu16* was adjacent to a major QTL, *Hdlq17*, for high-density lipoprotein (HDL) cholesterol (LOD: 6.31, peak: 19.1 cM). A congenic strain with a donor chromosomal region harboring *Bglu16* and *Hdlq17* on the *Apoe*^−/−^ background showed elevations in plasma glucose and HDL levels. Fasting glucose levels were significantly correlated with non-HDL cholesterol and triglyceride levels, especially on the Western diet, but only marginally correlated with HDL levels in F_2_ mice.

**Conclusions:**

We have demonstrated a correlative relationship between fasting glucose and plasma lipids in a segregating F_2_ population under hyperlipidemic conditions, and this correlation is partially due to genetic linkage between the two disorders.

## Introduction

Dyslipidemia, characterized by elevations in plasma triglyceride and LDL cholesterol levels and reductions in HDL cholesterol levels, frequently occurs with hyperglycemia as part of the metabolic syndrome, which also includes abdominal obesity, insulin resistance, and hypertension [1]. Although the nature for the close association between dyslipidemia and hyperglycemia is not well understood, pleiotropic effects of genetic mutants or variants affecting both traits appear to play a role. Indeed, a few rare genetic mutations involving *ABCA1* [2], *LIPE*[3], *LPL*[4], or *LRP6* [5]cause both dyslipidemia and hyperglycemia. Genome-wide association studies (GWAS) have identified a number of common variants associated with variations in plasma lipids[6][7] and fasting plasma glucose [8][9][10]. Over a dozen of them are associated with both traits at the genome-wide significance level (http://www.genome.gov/GWAStudies/). Unexpectedly, half of them, including *CETP*, *MLXIPL*, *PLTP*, *GCKR*, *APOB*, *APOE-C1-C2*, *CYP7A1*, and *TIMD4*, have exhibited opposite allelic effect on plasma lipid and glucose levels [11], a finding that is incontrary to the positive correlations observed in the clinical situation. Furthermore, it is quite challenging to establish causality between a common variant and a complex trait in humans due to small gene effect, complex genetic structure, and environmental influences.

One approach to the problems encountered in human genetic studies is to use inbred strains of mice differing in glucose and lipid profile. Apolipoprotein E-deficient (*Apoe*^−/−^) mice develop spontaneous dyslipidemia on a low fat chow diet, with elevated non-HDL cholesterol levels and reduced HDL levels [12][13]. Feeding a high fat diet aggravates dyslipidemia. Moreover, these mice develop all phases of atherosclerotic lesions seen in humans [14][15][16][17][18]. We have found that *Apoe*^−/−^ mice with the C57BL/6J, C3H/HeH, SM/J (SM) or SWR/J genetic background develop significant hyperglycemia when fed a Western diet but become resistant when transferred on to the BALB/cJ (BALB) background [19][20][16]. In the present study, we performed quantitative trait locus (QTL) analysis using a male cohort derived from BALB-*Apoe*^−/−^ and SM-*Apoe*^−/−^ mice to find potential genetic connections between plasma glucose and lipid traits.

## Methods

All procedures were carried out in accordance with current National Institutes of Health guidelines and approved by the University of Virginia Animal Care and Use Committee (Assurance #A3245-01, Animal Protocol #3109).

### Mice

BALB and SM *Apoe*^-/-^ mice were created using the classic congenic breeding strategy, as described[16]. BALB-*Apoe*^-/-^ mice were crossed with SM-*Apoe*^-/-^mice to generate F_1_s, which were intercrossed by brother-sister mating to generate a cohort of F_2_ mice. Mice were weaned at 3 weeks of age onto a rodent chow diet. At 8 weeks of age, male F_2_ mice were started on a Western diet containing 21% fat, 34.1% sucrose, 0.15% cholesterol, and 19.5% casein by weight (Harlan Laboratories, TD 88137) and maintained on the diet for 5 weeks.

### Measurements of plasma glucose and lipid levels

Mice were bled twice: once before the start of the Western diet and once at the time of euthanasia. Mice were fasted overnight before blood was drawn from the retro-orbital venous plexuswith the animals under isoflurane anesthesia. Plasma glucose was measured with a Sigma glucose (HK) assay kit, as reported [21]. Total cholesterol, HDL cholesterol, and triglyceride were measured using Thermo DMA (Louisville, CO) assay kits[13]. Non-HDL cholesterol was calculated as the difference between total and HDL cholesterol.

### Genotyping

Genomic DNA was isolated from the tails of mice by using the phenol/chloroform extraction and ethanol precipitation method. F_2_ mice were genotyped by the Jackson Laboratory Genotyping Services using mouse strain-specific SNP arrays. DNA samples from the two parental strains and their F_1_s served as controls. 144 SNPs and 225 F_2_ mice were included for QTL analysis.

### Studies with congenic mice

Construction of a congenic strain in which a chromosome 9 segment from 5 to 61 cM was transferred from C3H/HeJ-*Apoe*^-/-^ mice onto the C57BL/6J-*Apoe*^-/-^ background was previously reported [22]. Male congenic and C57BL/6J-*Apoe*^-/-^ control mice were started with the Western diet at 6 weeks of age and maintained on the diet for 12 weeks. Blood samples were collected from overnight fasted mice before and after 12 weeks of Western diet.

### Statistical analysis

QTL analysis was performed using the standard analysis softwared J/qtl and Map Manager QTX as we previously reported[19][23][24]. One thousand permutations of trait values were run to define the genome-wide LOD (logarithm of odds) score threshold for significant or suggestive linkage of each trait. Loci that exceeded the 95th percentile of the permutation distribution were considered significant (*P*<0.05) and those exceeding the 37th percentile were suggestive (*P*<0.63). Student's unpaired t test was used to determine statistical significance between congenic and control mice in trait values.

## Results

### Trait value distributions

**Fig 1:**
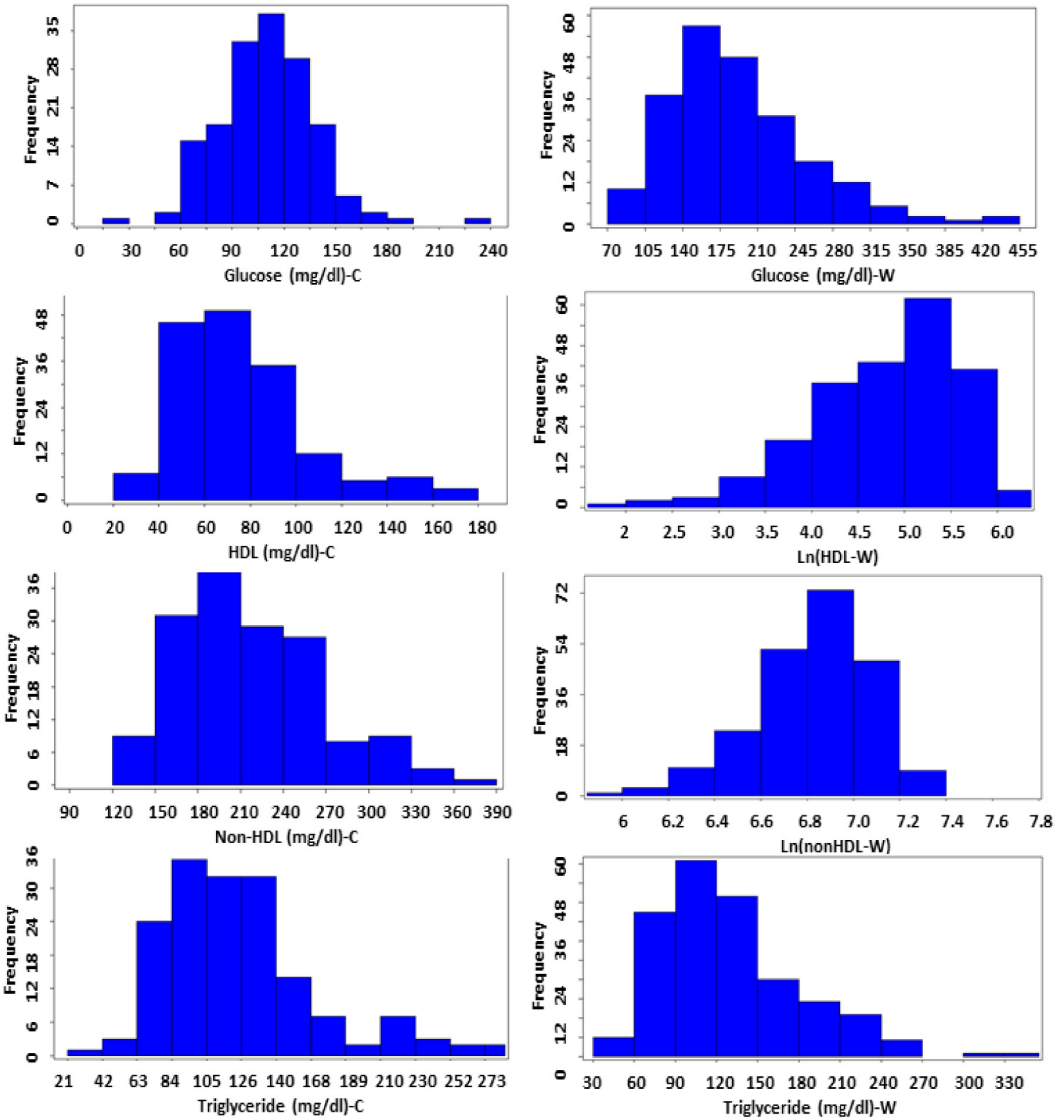
Distributions of trait values for fasting plasma glucose, HDL, non-HDL cholesterol and triglyceride of 225 male F_2_ mice derived from an intercross between BALB-*Apoe*^−/−^ and SM-*Apoe*^−/−^ mice. Values of HDL and non-HDL cholesterol levels on the Western diet were transformed to natural logarithm (Ln). Blood was collected once before (left panel) and once after 5 weeks of Western diet (right panel). Graphs were created using a plotting function of J/qtl software. C, chow diet; W, Western diet.

Values of fasting plasma glucose and triglyceride levels of F_2_ mice on both chow and Western diets and of HDL and non-HDL cholesterol levels on the chow diet are normally or approximately normally distributed (Fig 1).Values of Ln (natural logarithm)-transformed HDL and non-HDL cholesterol levels on the Western diet approach the normal distribution. These data were analyzed using J/qtl software to search for QTLs affecting the traits. Loci with a genome-wide suggestive or significant *P* value are presented in Table 1.

**Table 1:** Significant and suggestive QTLs for plasma glucose and lipid levels in male F_2_ mice derived from BALB.*Apoe*^-/-^ and SM.*Apoe*^-/-^ mice.Locus name.

	Diet	Chr	Trait	LOD^a^	Peak (cM)	95%CI^b^	P value^c^	High allele	Mode of inheritance^d^
***Bglu16***	Chow	9	Glucose	**6.463**	12.70	9.135–17.135	0.001	B	Additive
*Bglu8*	chow	15	Glucose	3.263	3.658	1.66–57.04	0.151	B	Recessive
*Bglu10*	Chow	18	Glucose	2.279	15.911	9.91–27.91	0.606	S	Additive
	Western	X	Glucose	3.069	68.386	66.386–70.386	0.425	S	
*Hdlq5*	chow	1	HDL	2.992	82.442	72.14–90.14	0.463	B	Additive
*Hdlq18*	chow	12	HDL	3.428	27.877	23.88–33.88	0.321	S	Recessive
*Hdlq60*	Chow	X	HDL	2.653	72.386	20.39–74.80	0.602	B	
*Cq1*	Western	1	HDL	3.236	76.141	66.14–84.14	0.193	B	Additive
***Hdlq17*, *Cq4***	Western	9	HDL	**6.311**	19.134	15.14–27.14	0.001	B	Additive
*Hdlcl2*	Western	13	HDL	2.406	7.12	2.319–67.427	0.606	B	Additive
*Hdlq45*	Western	15	HDL	2.509	1.658	1.66–57.04	0.54		Heterosis
*Hdlq56*	Western	17	HDL	2.602	60.903	39.17–60.90	0.576		Heterosis
*Nhdlq13*	Western	1	Non-HDL	3.942	60.141	54.14–66.14	0.081	B	Dominant
*Nhdlq9*	Western	15	Non-HDL	3.52	41.658	31.66–51.66	0.132	S	Additive
***Tgq35***	*Chow*	X	Triglyceride	3.063	62.386	66.386–72.386	0.587	B	
*Tgq9*	Western	1	Triglyceride	3.239	41.777	34.14–86.14	0.255	B	Additive
*Tglq1*	Western	1	Triglyceride	2.74	80.1	69.0–90.0	<0.63	B	Additive
***Tgq35***	Western	X	Triglyceride	3.251	66.386	66.39–72.39	0.248	B	

^a^ LOD scores were obtained from genome-wide QTL analysis using J/qtl software. The significant LOD scores were highlighted in bold. The suggestive and significant LOD score thresholds were determined by 1,000 permutation tests for each trait. Suggestive and significant LOD scores were 2.249 and 3.778, respectively, for glucose on chow diet; 2.545 and 5.211 for glucose on Western diet; 2.588 and 5.713 for HDL cholesterol, 2.348 and 4.243 for non-HDL cholesterol, and 2.655 and 6.568 for triglyceride on the chow diet; 2.438 and 4.377 for HDL, 2.348 and 4.243 for non-HDL, and 2.438 and 4.377 for triglyceride on the Western diet.

^b^ 95% Confidence interval in cM defined by a whole genome QTL scan.

^c^ The p-values reported represent the level of genome-wide significance as they were generated base d on genome-wide permutation tests.

^d^ Mode of inheritance was defined according to allelic effect at the nearest marker of a QTL.

### Fasting glucose levels

**Fig 2:**
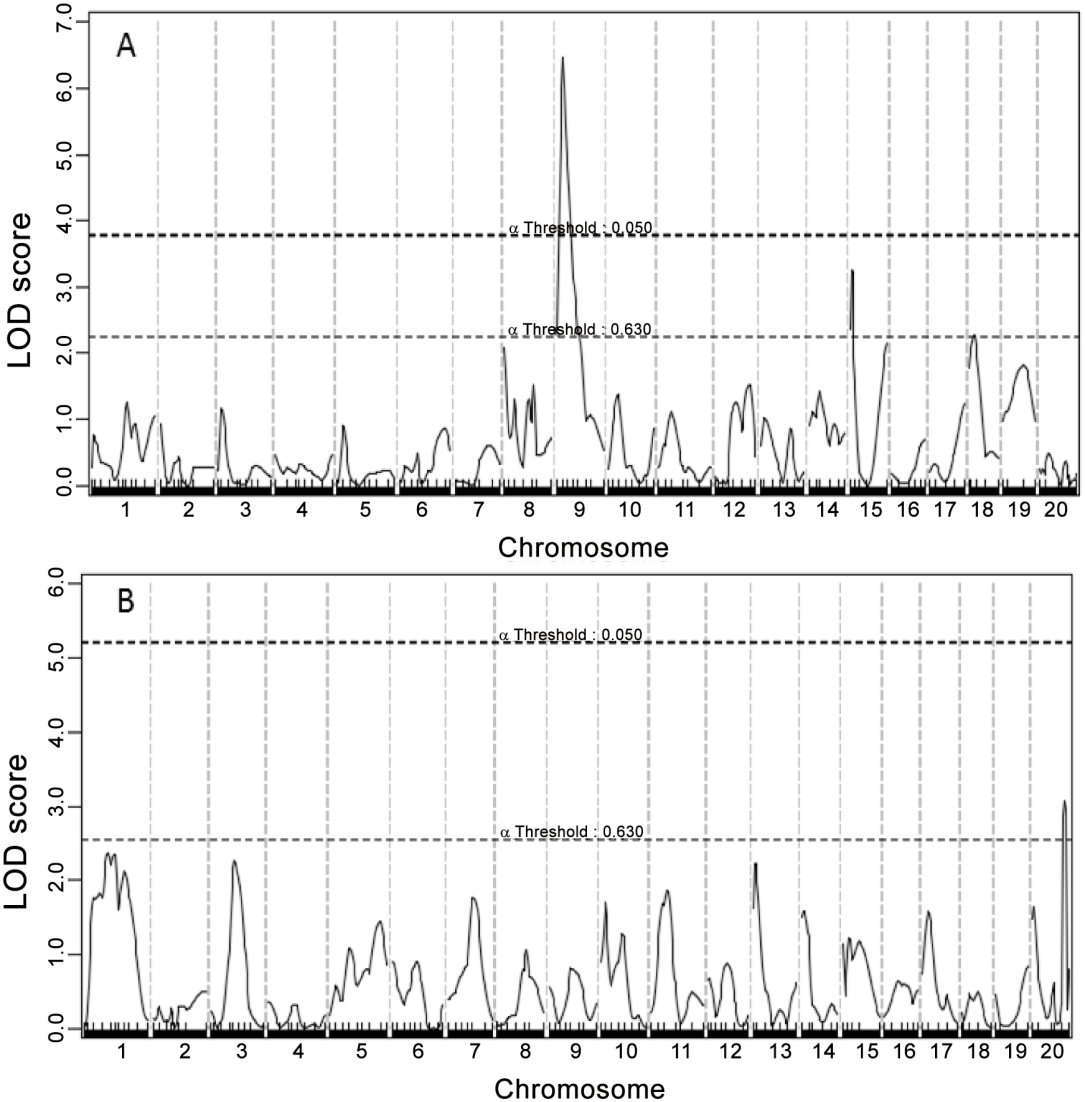
Genome-wide scans to search for main effect loci influencing fasting plasma glucose levels. (A) male F_2_ mice were fed a chow diet. (B) mice were fed a Western diet. Chromosomes 1 through X are represented numerically on the X-axis. The Y-axis represents the LOD score. Two horizontal dashed lines denote genome-wide empirical thresholds for suggestive (*P* = 0.63) and significant (*P* = 0.05) linkage.

A genome-wide scan for main effect QTL revealed a highly significant QTL in the proximal region of Chr9 for fasting glucose levels when mice were fed the chow diet (12.7cM, LOD:6.463) (Fig 2 and Table 1) (original genotype and phenotype data used for QTL analysis are provided in Table A in S1 text). This locus is overlapping in position with *Bglu16*, recently mapped in female F_2_ mice derived from BALB and SM Apoe^-/-^ mice. Two suggestive loci, located on Chr15 and Chr18, for fasting glucose were also detected when the cross were on the chow diet. The Chr15 locus replicates *Bglu8* and the Chr18 locus replicates *Bglu10*, initially mapped in a NZB/B1NJ x NZW/LacJ intercross [25]. When F_2_ mice were fed the Western diet, a suggestive locus for fasting glucose was detected in the distal region of ChrX (68.38 cM, LOD: 3.069). This locus was novel. Inheritance of BALB alleles conferred an increased glucose level for the Chr9 and Chr15 QTLs while inheritance of SM alleles conferred increased glucose levels for Chr18 and ChrX QTLs (Table 2).

**Table 2:** Allelic effects in different QTLs on fasting plasma glucose and lipid levels of male F2 mice derived from BALB and SM Apoe−/− mice.

Locus name	Chr	Trait	LOD	Peak (cM)	Closest marker	BB	SS	SB
***Bglu16***	9	Glucose-C	**6.463**	12.70	rs3704408	126.5 ± 28.9 (n = 49)	98.3 ± 21.7 (n = 48)	108.3 ± 21.8 (n = 59)
*Bglu8*	15	Glucose-C	3.263	3.658	rs3687235	123.2 ± 32.2 (n = 41)	105.2 ± 22.5 (n = 40)	107.2 ± 22.9 (n = 75)
*Bglu10*	18	Glucose-C	2.279	15.911	rs3705122	102.7 ± 22.0 (n = 30)	123.1 ± 23.0 (n = 33)	109.0 ± 27.6.0 (n = 95)
	X	Glucose-W	3.069	68.386	rs3723498	190.6 ± 66.8 (n = 101)	193.2 ± 63.2 (n = 117)	
*Hdlq5*	1	HDL-C	2.992	82.442	rs3654101	88.6 ± 34.6 (n = 41)	63.8 ± 23.7 (n = 39)	76.0 ± 24.4 (n = 77)
*Hdlq18*	12	HDL-C	3.428	27.877	rs3707048	72.8 ± 26.0 (n = 44)	93.7 ± 35.0 (n = 35)	69.7 ± 22.8 (n = 76)
*Hdlq60*	X	HDL-C	2.653	72.386	rs3725586	77.8 ± 30.7 (n = 71)	74.8 ± 26.5 (n = 87)	
*Cq1*	1	HDL-W	3.236	76.141	rs3654101	207.3 ± 119.3 (n = 58)	109.0 ± 77.9 (n = 59)	167.6 ± 105.5 (n = 99)
*Hdlq17*	9	HDL-W	**6.311**	19.134	rs3023205	178.9 ± 110.1 (n = 63)	142.6 ± 108.2 (n = 58)	155.7 ± 103.1 (n = 98)
*Hdlcl2*	13	HDL-W	2.406	7.12	rs13481718	188.7 ± 128.6 (n = 54)	118.0 ± 88.5 (n = 51)	170.2 ± 100.8 (n = 114)
*Hdlq45*	15	HDL-W	2.509	1.658	rs3687235	150.3 ± 103.3 (n = 59)	142.0 ± 107.5 (n = 54)	172.6 ± 105.0 (n = 103)
*Hdlq56*	17	HDL-W	2.602	60.903	rs3707114	149.0 ± 105.9 (n = 64)	154.0 ± 112.2 (n = 58)	166.8 ± 101.2 (n = 97)
*Nhdlq13*	1	Non-HDL-W	3.942	60.141	rs13459053	982.5 ± 213.2 (n = 56)	855.7 ± 195.6 (n = 43)	975.4 ± 213.5 (n = 119)
*Nhdlq9*	15	Non-HDL-W	3.52	41.658	rs3667910	878.4 ± 228.9 (n = 52)	1008.4 ± 229.5 (n = 48)	958.4 ± 196.8 (n = 120)
***Tgq35***	X	Triglyceride-C	3.063	62.386	rs3723498	133.1 ± 48.7 (n = 70)	120.9 ± 45.8 (n = 89)	
*Tgq9*	1	Triglyceride-W	3.239	41.777	rs3022821	150.4 ± 61.1 (n = 42)	114.7 ± 37.1 (n = 55)	130.9 ± 50.2 (n = 120)
*Tglq1*	1	Triglyceride-W	2.74	80.1	rs3654101	149.8 ± 60.4 (n = 59)	119.8 ± 43.9 (n = 59)	127.7 ± 46.3 (n = 99)
***Tgq35***	X	Triglyceride-W	3.251	66.386	rs3723498	133.8 ± 55.6 (n = 101)	126.9 ± 46.1 (n = 118)	

Data are mean ± SD. The units for these measurements are mg/dL for plasma glucose or lipid levels. The number in the brackets represents the number of progeny with a specific genotype at a peak marker. The significant QTLs and their LOD scores were highlighted in bold. Chr, chromosome; LOD, logarithm of odds; C, chow diet; W, Western diet; BB, homozygous BALB allele; SS, homozygous SM allele; SM, heterozygous allele.

### Fasting lipid levels

**Fig 3:**
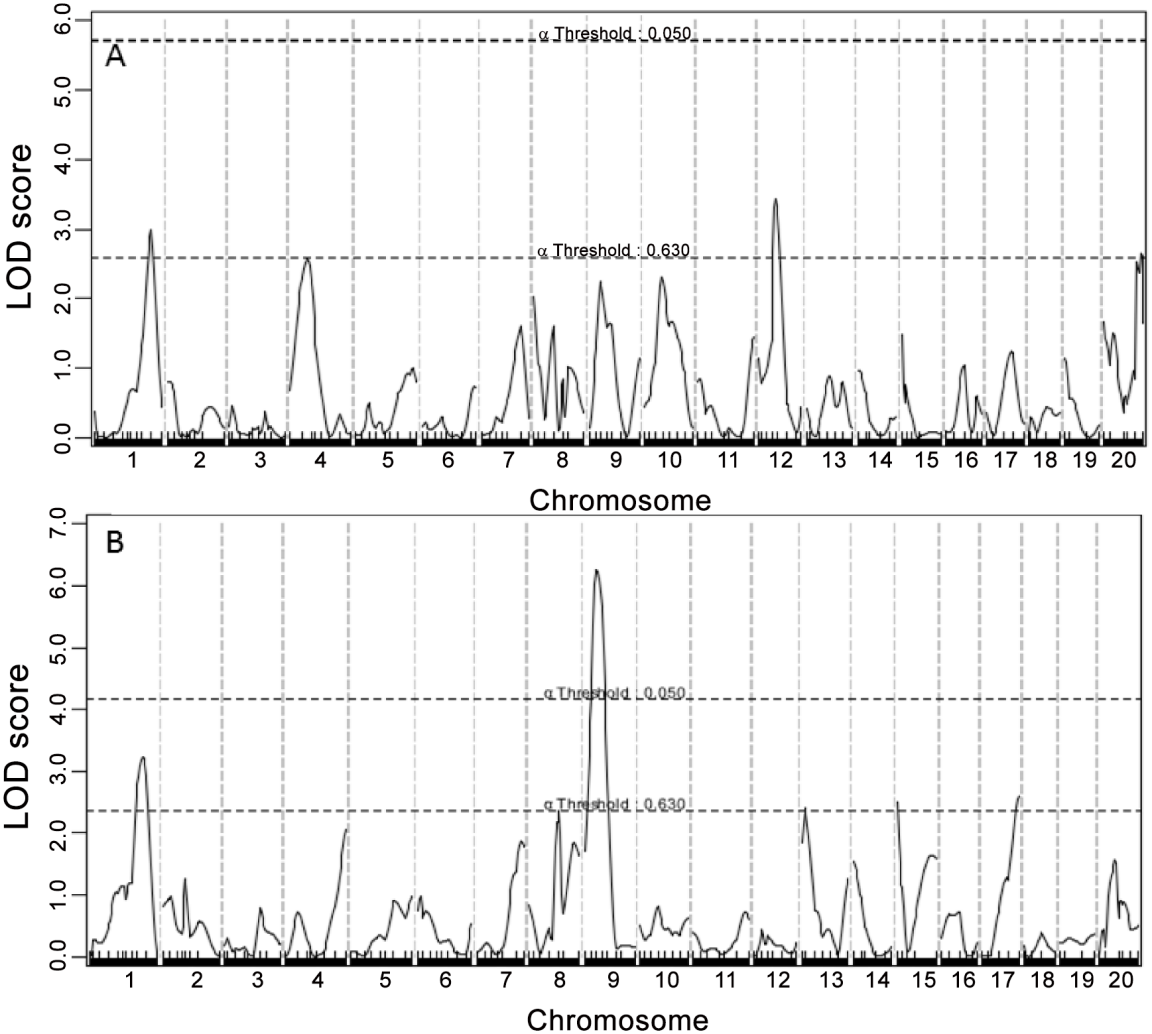
Genome-wide scans to search for loci influencing HDL cholesterol levels. (A) male F_2_ mice were fed a chow diet. (B) Mice were fed a Western diet.

**Fig 4:**
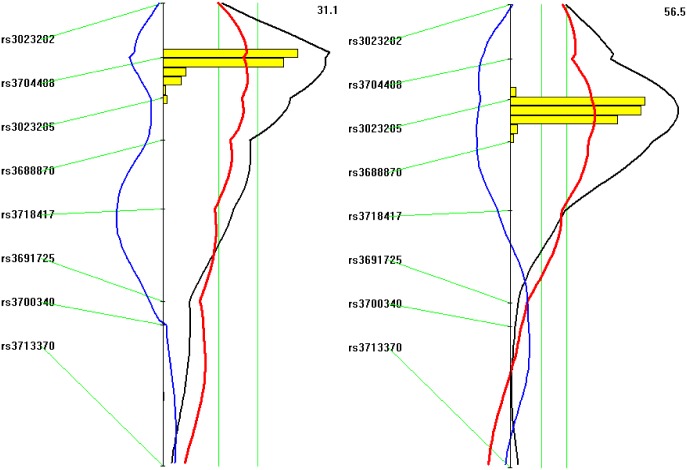
Interval mapping graphs for fasting glucose (left panel) and HDL (right panel) on chromosome 9. The histogram in the plot estimates the confidence interval for a QTL. Note the difference in position between the two QTLs. Two green vertical lines represent genome-wide significance thresholds for suggestive or significant linkage (*P* = 0.63 and *P* = 0.05, respectively). Black plots reflect the LOD score calculated at 1-cM intervals, the red plot represents the effect of BALB alleles, and the blue plot represents the effect of SM alleles.

**Fig 5:**
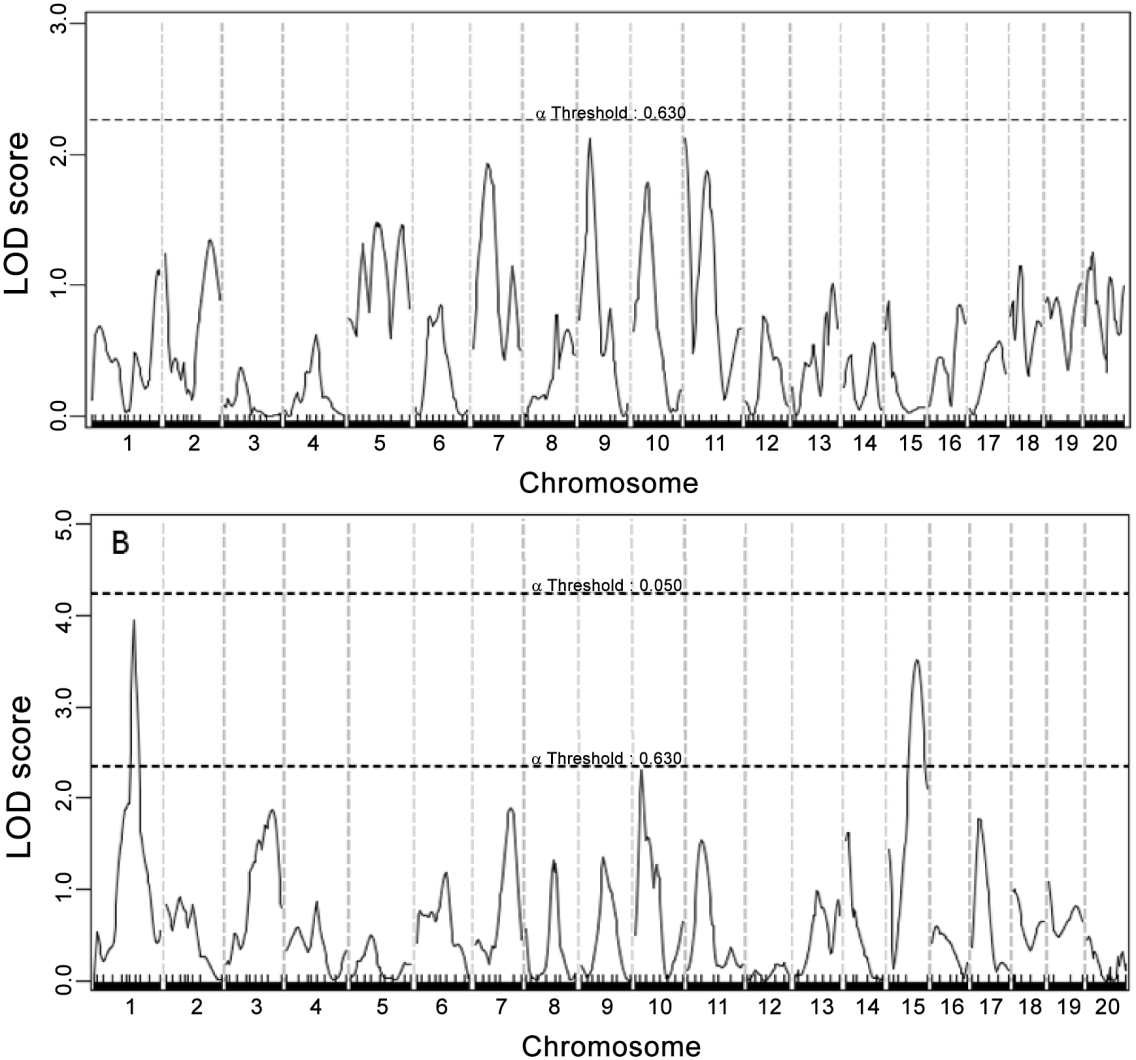
Genome-wide scans to search for loci influencing non-HDL cholesterol levels. (A) F_2_ mice were fed a chow diet. (B) Mice were fed a Western diet. Two loci on chromosomes 1 and 15 were identified for non-HDL cholesterol levels under the Western diet.

Genome-wide scans for main effect QTLs detected multiple loci forHDL, non-HDL cholesterol, and triglyceride levels (Figs 3, 4 and 5, Table 1). For HDL, 3 suggestive QTLs, located on Chr1, Chr12 and Chr20, were found on the chow diet and 5 QTLs, located on Chr1, 9, 13, 15 and 17, were found on the Western diet. The Chr9 QTL peaked at 19.13 cM and had a highly significant LOD score of 6.31 (Table 1). This QTL is overlapping in position with *Hdlq17*, mapped in female B6x129S1/SvImJ F_2_ mice [26]. Though partially overlapping, the position of this QTL was noticeably different from that of *Bglu16* (Fig 4). The Chr1 locus replicated *Cq1* and *Hdlq5*, which have been mapped in numerous crosses[27]. The Chr13 QTL replicated *Hdlcl2*, initially mapped in (PERA/EiJ x B6-*Ldlr*)) x B6-*Ldlr* backcross [28]. The Chr15 QTL replicated *Hdlq45*, previously mapped in a B6 x A/J intercross[29]. The Chr17 locus replicated *Hdlq56*, mapped in a B6 x 129 intercross [30].

For non-HDL, 2 QTLs on Chr1 and Chr15 were detected when mice were fed the Western diet. The Chr1 QTL peaked at 60.14 cM and had a LOD score of 3.94 (Fig 5 and Table 1). This QTL replicated *Nhdlq13*, mapped in a B6 x C3H Apoe^-/-^ intercross [31]. The QTL on Chr15 peaked at 41.66 cM and had a LOD score of 3.52. It replicated *Nhdlq9*, mapped previously in PERA/EiJ X DBA/2J and B6-Apoe^-/-^ X C3H-Apoe^-/-^ intercrosses[32][33].

**Fig 6:**
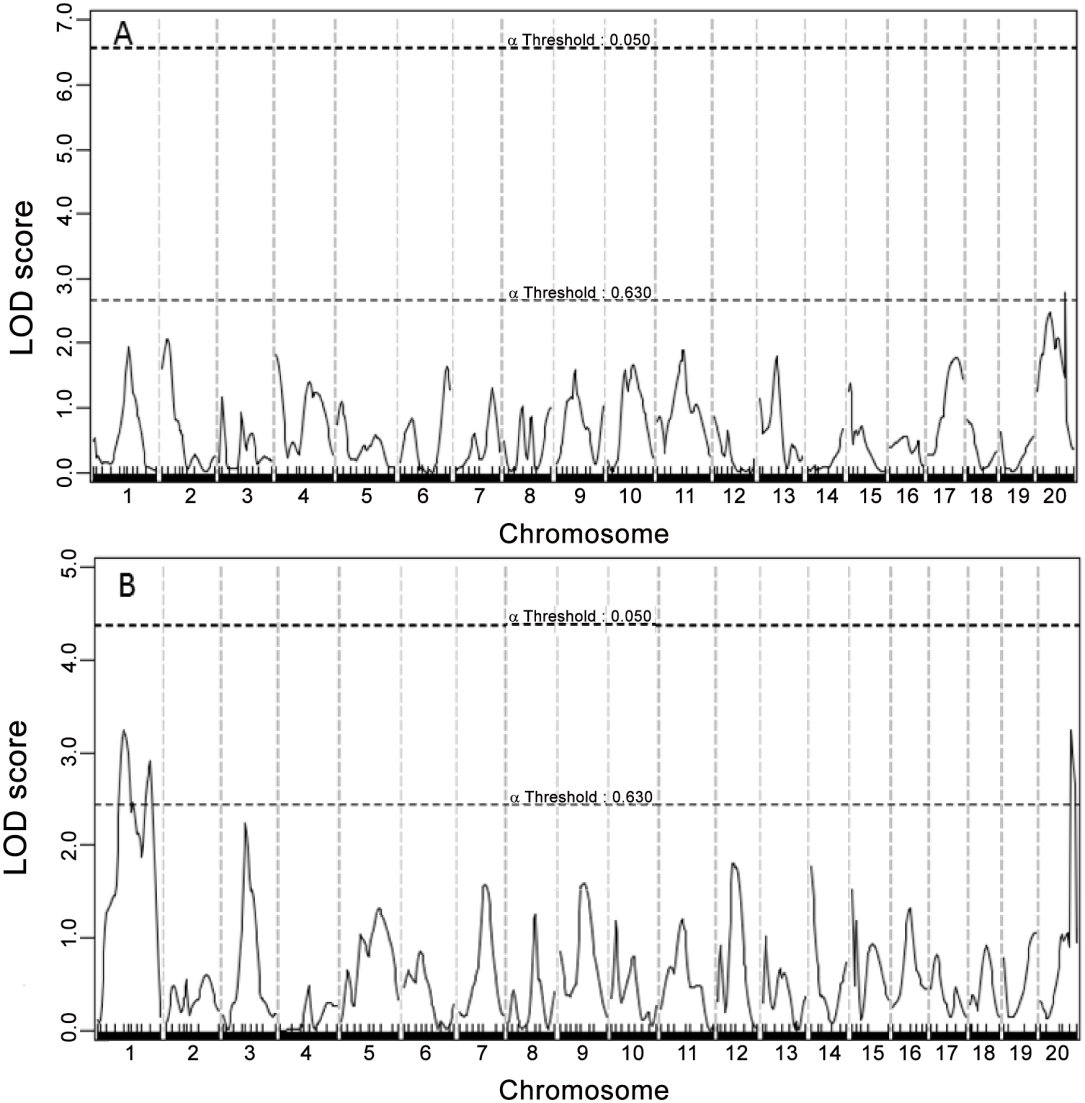
Genome-wide scans to search for loci influencing triglyceride levels. (A) F_2_ mice were fed a chow diet. (B) Mice were fed a Western diet. Three suggestive loci on chromosomes 1 and X were identified for triglyceride levels.

For triglyceride, 3 suggestive QTLs, located on Chr1 and ChrX, were detected when mice were fed the Western diet (Fig 6). The QTL on ChrX had a LOD score of 3.25 and peaked at 66.4 cM. This QTL was replicated on the chow diet and thus named *Tgq35*. The 2 suggestive QTLs on Chr1, peaked at 41.8 and 80.1 cM, replicated Tgq9 *and Tglq1*, respectively[34].

### Correlations between plasma glucose and lipid levels

**Fig 7:**
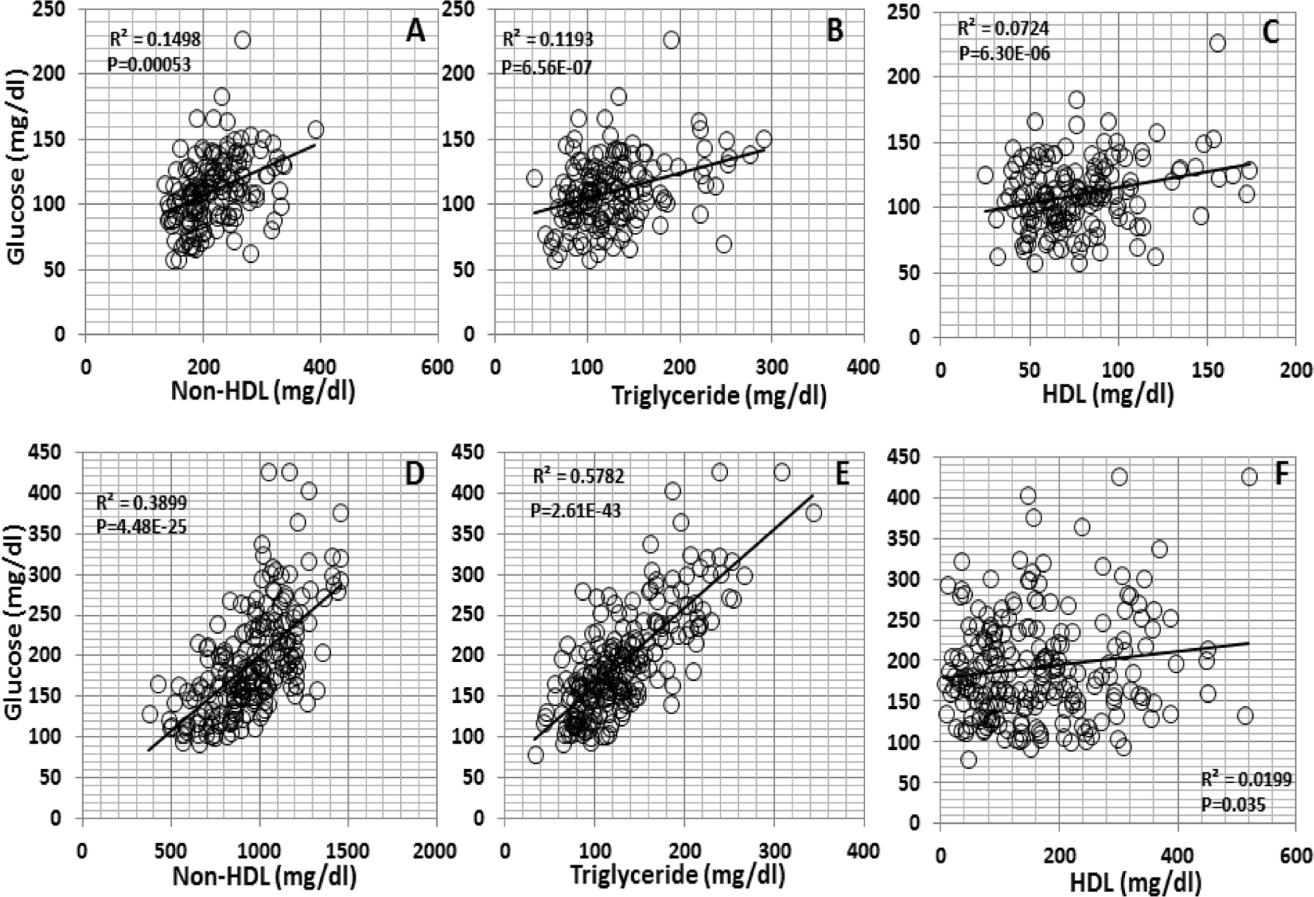
Correlations of fasting plasma glucose levels with plasma levels of HDL, non-HDL cholesteroland triglyceride. The F_2_ population were fed a chow (top row: A, B, C) or Western diet (bottom row: D, E, F). Each point represents values of an individual F_2_ mouse. The correlation coefficient (*R*^*2*^) and significance (*P*) are shown.

Correlations of fasting plasma glucose levels with plasma levels of HDL, non-HDL cholesterol or triglyceride were evaluated in the F_2_ population fed either chow or Western diet (Fig 7). Significant correlations of fasting glucose with non-HDL cholesterol and triglyceride were observed when mice were fed either chow (*R*^*2*^ = 0.1498 and *P* = 5.3E-4 for non-HDL; *R*^*2*^ = 0.1193 and *P* = 6.56E-7 for triglyceride) or Western diet (*R*^*2*^ = 0.3899 and *P* = 4.48E-25 for non-HDL; *R*^*2*^ = 5782 and *P* = 2.61E-43 for triglyceride). F_2_ mice with higher non-HDL cholesterol or triglyceride levels also had higher fasting glucose levels, especially on the Western diet. In contrast, HDL cholesterol levels were only marginally correlated with fasting glucose levels on either chow (*R*^*2*^ = 0.0724 and *P* = 6.3E-6) or Western diet (*R*^*2*^ = 0.0199 and *P* = 0.035).

### Confirmation of chromosome 9 QTLs

**Fig 8:**
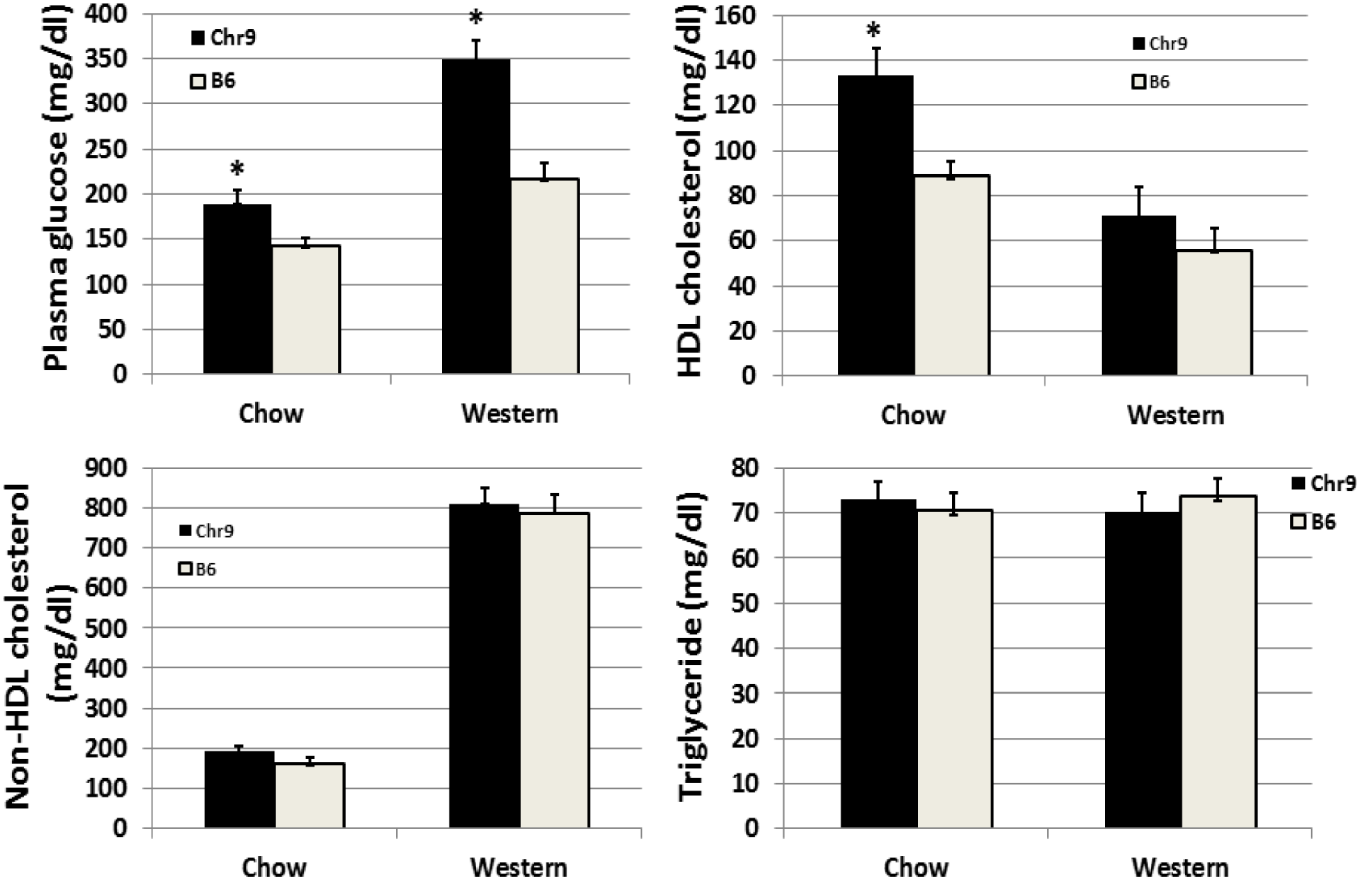
Comparison of male congenic and background control mice in fasting plasma glucose, HDL, non-HDL cholesterol, and triglyceride levels when fed a chow or Western diet. Congenic mice contained a donor C3H/HeJ chromosome 9 segment harboring *Bglu16* and *Hdlq17* on the C57BL/6J Apoe^-/-^ background. Results are means ± SE of 9 to 14 mice. * *P*< 0.05.

C3H/HeJ and BALB strains share essentially identical haplotype blocks for the chromosome 9 region harboring *Bglu16* and *Hdlq17* (10–30 cM), and also QTLs for fasting glucose and HDL have been mapped in this region using intercrosses derived from C3H/HeJ[19][35]. Thus, we used a congenic strain carrying a chromosomal region harboring *Bglu16* and *Hdlq17* from the C3H/HeJ donor strain to test QTL effects on fasting glucose and lipid profile. Male congenics had significantly higher fasting plasma glucose levels than C57BL/6 *Apoe*^−/−^ mice on either chow (189.1 ± 8.8 vs. 142.0 ± 15.2 mg/dl; *P* = 0.017) or Western diet (348.8 ± 19.0 vs. 215.9 ± 20.6 mg/dl; *P* = 0.00017) (Fig 8 and Table B in S1 text). HDL cholesterol levels were nearly 2-fold higher in congenics than in C57BL/6 *Apoe*^−/−^ mice on the chow diet (133.0 ± 12.0 vs. 88.4 ± 6.4 mg/dl; *P* = 0.0039). On the Western diet, HDL cholesterol levels were also higher in congenics (71.1 ± 12.5 vs. 55.6 ± 9.7 mg/dl), though the difference did not reach statistical significance (*P* = 0.339). In contrast, congenics were comparable with C57BL/6 *Apoe*^−/−^ mice in non-HDL cholesterol levels (chow: 191.5 ± 15.2 vs. 160.1 ± 16.5 mg/dl, *P* = 0.177; Western: 809.5 ± 40.7 vs. 784.2 vs. 46.8 mg/dl, *P* = 0.689) and triglyceride levels (chow: 73.1 ± 3.8 vs. 70.4 ± 3.9 mg/dl, *P* = 0.626; Western: 70.0 ± 4.5 vs. 73.7± 3.8 mg/dl, *P* = 0.543).

## Discussion

BALB *Apoe*^*-/-*^mice have much higher HDL and lower non-HDL cholesterol levels and are more resistant to development of type 2 diabetes compared to SM*Apoe*^*-/-*^mice[16][20]. In this study, we performed QTL analysis using a male F_2_ cohort derived from the two *Apoe*^*-/-*^mouse strains to investigate genetic connections between glucose and lipid-related traits. One major QTL for fasting glucose, *Bglu16*, is immediately adjacent but not coincident with a major QTL for HDL cholesterol, *Hdlq17*, on proximal chromosome 9. The presence of these two QTLs was confirmed with a congenic strain. Moreover, strong correlations of fasting glucose with non-HDL and triglyceride levels were observed in F_2_ mice when fed the Western diet.

In this study, one significant QTL and three suggestive QTLs have been identified to influence glucose homeostasis under fasting conditions. The significant QTL on proximal chromosome 9 is coincident with *Bglu16*, recently mapped in a female intercross between BALB and SM Apoe^-/-^ mice. A suggestive QTL for fasting glucose has also been mapped to this position in a C57BL/6 x BALB Apoe^-/-^intercross[21]. For all three intercrosses, inheritance of BALB alleles at the locus contributed to increased fasting glucose levels. A locus for glucose-stimulated insulin secretion has been mapped to this position in a C57BL/6J x C3H/HeJ intercross [36].The suggestive QTL on chromosome 15 is close to *Bglu8*, mapped in a NZB x NZW intercross [25]. Linkages close to this locus have been detected in two other crosses involving BALB mice but inheritance of BALC alleles was associated with reduced fasting glucose levels [21][37]. The QTL on chromosome 18 is coincident with *Bglu10*, mapped in a NZB x NZW intercross [25].

All QTLs identified for plasma lipids confirm those mapped in previous studies except for the one on X chromosome for triglyceride that is new and named *Tgq30*. This QTL occurred with a suggestive LOD score under both chow and Western diet feeding conditions. It is considered appropriate to name a suggestive QTL if it has been repeatedly observed [38]. The major QTL for HDL is coincident with *Hdlq17*, mapped in a C57BL/6 x 129S1/SvImJ female intercross [26]. This QTL has been replicated in multiple crosses, including B6 x 129, B6 x CAST/EiJ, B6-*Apoe*^-/-^ x C3H-*Apoe*^-/-^, and B6-*Apoe*^-/-^ x BALB-*Apoe*^-/-^ intercrosses [33][39][40][41][30][31]. We conducted haplotype analysis for this QTL and narrowed candidates down to two dozen genes (Table C in S1 Text). These candidate genes contain one or more non-synonymous SNPs in the coding regions or SNPs in the upstream regulatory region that are shared by the high allele strains but are different from the low allele strains at the QTL. Among them, *Ubash3b*, *Phldb1*, *Sorl1*, *Sik3*, and *Apoa1* have been shown to be associated with variations in total, HDL cholesterol or triglyceride levels in humans (http://www.ebi.ac.uk/gwas/home). Linkage close to this locus has also been detected in a female intercross derived from BALB and SM Apoe^-/-^ mice but BALB alleles were associated with to reduced HDL levels [42]. The opposite allelic effect on HDL in the male vs. female crosses suggests that two or more genes in this region contributed to the trait.

As BALB and C3H/HeJ strains share essentially identical haplotype blocks for the chromosomal region harboring *Bglu16* and *Hdlq17* and as QTLs for fasting glucose and HDL have been mapped to this region in crosses derived from C3H/HeJ mice [19][35], we used a congenic strain carrying the C3H/HeJ chromosome 9 donor alleles to confirm the presence of the two QTLs. However, as the congenic strain carries a chromosomal segment much longer than the confidence interval of *Bglu16* and *Hdlq17*, other QTLs in the congenic region might also contribute to the QTL effects observed in the congenic mice.

We have observed positive correlations of fasting glucose levels with non-HDL cholesterol and triglyceride levels in the F2 population under either feeding condition. The correlations were extremely high when mice developed significant dyslipidemia on the Western diet. Similar findings have been observed in other crosses derived from Apoe^-/-^ mouse strains[21] and humans [11][43]. Emerging human studies have also shown associations of non-HDL cholesterol and ApoB with incident type 2 diabetes [44],[45],[46]. Despite the strong correlations, only one suggestive QTL for fasting glucose was coincident with one QTL for triglyceride on chromosome X and there was no coincident QTL between glucose and non-HDL traits.

We observed a slight but positive correlation between HDL cholesterol and fasting glucose levels in male F_2_ mice on either chow or Western diet. This finding is in contrast with the negative correlation between the two traits in a female F_2_ cohort derived from the same parental strains[42]. Prospective human studies have shown that HDL is inversely correlated with the risk of type 2 diabetes [47][48]. HDL can increase insulin secretion from β-cells, improve insulin sensitivity of the target tissues, and accelerate glucose uptake by muscle due to its diverse functions, including cholesterol efflux and reverse cholesterol transport, anti-oxidation, anti-inflammation and activation of the AMP-activated protein kinase[49][50]. The positive correlation between HDL and fasting glucose observed in this study may suggest that HDL has lost its anti-diabetic function. Indeed, under pathological conditions such as the acute phase response and chronic inflammatory diseases, HDL undergoes qualitative changes in both components and structure and can lose protective function[51]. The observed correlation between HDL and fasting glucose could also be derived from the genetic effect of two closely linked QTLs with each affecting one trait, like *Bglu16* and *Hdlq17*. The reasons for the discrepancy between male and female F_2_ mice in the correlations are unknown. Multiple factors could contribute: First, female mice were fed the western diet for 12 weeks starting at 6 weeks of age while males were fed the diet for 5 weeks starting at 8 weeks of age. Second, male F_2_s had higher glucose levels (chow: 110 vs 99, Western: 191 vs 147 mg/dl) than their female counterparts, suggesting that males are more susceptible to diet-induced type 2 diabetes. Finally, sex differences in metabolic traits have been observed in humans and mice [52][53].

Dyslipidemia and hyperglycemia are integral components of metabolic syndrome, a group of risk factors that increase risk for cardiovascular disease and type 2 diabetes. We have identified multiple loci contributing to dyslipidemia and hyperglycemia from a male F_2_cohort. One major QTL for fasting glucose, *Bglu16*, is adjacent to *Hdlq17*, a QTL for HDL on chromosome 9. The strong correlations of fasting glucose with non-HDL cholesterol and triglyceride support the hypothesis that dyslipidemia plays a causative role in the development of type 2 diabetes [54].

## Supporting Information

S1 Text Supporting tables: genotypic and phenotypic data used for quantitative trait locus (QTL) analysis, characterization of congenic strains, and haplotype analysis. (XLSX)
